# Inflammatory Trajectory of Type 2 Diabetes: Novel Opportunities for Early and Late Treatment

**DOI:** 10.3390/cells13191662

**Published:** 2024-10-08

**Authors:** Valeria Pellegrini, Rosalba La Grotta, Francesca Carreras, Angelica Giuliani, Jacopo Sabbatinelli, Fabiola Olivieri, Cesare Celeste Berra, Antonio Ceriello, Francesco Prattichizzo

**Affiliations:** 1 IRCCS MultiMedica, Via Fantoli 16/15, 20138 Milan, Italy; valeria.pellegrini@multimedica.it (V.P.); rosalba.lagrotta@multimedica.it (R.L.G.);; 2 Cardiac Rehabilitation Unit of Bari Institute, Istituti Clinici Scientifici Maugeri IRCCS, 70124 Bari, Italy; 3 Department of Clinical and Molecular Sciences (DISCLIMO), Università Politecnica delle Marche, 60127 Ancona, Italy; j.sabbatinelli@staff.univpm.it (J.S.); f.olivieri@univpm.it (F.O.); 4 Clinic of Laboratory and Precision Medicine, IRCCS INRCA, 60127 Ancona, Italy; 5 Advanced Technology Center for Aging Research, IRCCS INRCA, 60127 Ancona, Italy; 6 Department of Endocrinology, IRCCS Multimedica, 20138 Milan, Italy; cesare.berra@multimedica.it

**Keywords:** inflammaging, obesity, senescence, metabolic memory, epigenetics, trained immunity, IL-6, hs-CRP, GLP-1RA, SGLT-2i, colchicine, canakinumab

## Abstract

Low-grade inflammation (LGI) represents a key driver of type 2 diabetes (T2D) and its associated cardiovascular diseases (CVDs). Indeed, inflammatory markers such as hs-CRP and IL-6 predict the development of T2D and its complications, suggesting that LGI already increases before T2D diagnosis and remains elevated even after treatment. Overnutrition, unhealthy diets, physical inactivity, obesity, and aging are all recognized triggers of LGI, promoting insulin resistance and sustaining the pathogenesis of T2D. Once developed, and even before frank appearance, people with T2D undergo a pathological metabolic remodeling, with an alteration of multiple CVD risk factors, i.e., glycemia, lipids, blood pressure, and renal function. In turn, such variables foster a range of inflammatory pathways and mechanisms, e.g., immune cell stimulation, the accrual of senescent cells, long-lasting epigenetic changes, and trained immunity, which are held to chronically fuel LGI at the systemic and tissue levels. Targeting of CVD risk factors partially ameliorates LGI. However, some long-lasting inflammatory pathways are unaffected by common therapies, and LGI burden is still increased in many T2D patients, a phenomenon possibly underlying the residual inflammatory risk (i.e., having hs-CRP > 2 mg/dL despite optimal LDL cholesterol control). On the other hand, selected disease-modifying drugs, e.g., GLP-1RA, seem to also act on the pathogenesis of T2D, curbing the inflammatory trajectory of the disease and possibly preventing it if introduced early. In addition, selected trials demonstrated the potential of canonical anti-inflammatory therapies in reducing the rate of CVDs in patients with this condition or at high risk for it, many of whom had T2D. Since colchicine, an inhibitor of immune cell activation, is now approved for the prevention of CVDs, it might be worth exploring a possible therapeutic paradigm to identify subjects with T2D and an increased LGI burden to treat them with this drug. Upcoming studies will reveal whether disease-modifying drugs reverse early T2D by suppressing sources of LGI and whether colchicine has a broad benefit in people with this condition.

## 1. Introduction

Low-grade inflammation (LGI) is a hallmark of type 2 diabetes mellitus (T2D), being etiopathogenically linked to the whole trajectory of the disease, from the inception of metabolic imbalances to the development of complications [1,2]. However, the clinical relevance or utility of this phenomenon in different stages of T2D is still questioned, given that anti-inflammatory treatments have not been proven effective in reducing the incidence of T2D and have a limited and transitory effect on glycemic control [3]. On the contrary, four trials employing two anti-inflammatory drugs, canakinumab and colchicine, demonstrated a reduction in the incidence of cardiovascular diseases (CVDs) in patients at high risk for or with a previous CVD [4,5,6,7]. Of note, a consistent number of patients enrolled in such trials have T2D or prediabetes, and thus, these conditions might represent an indication for the introduction of anti-inflammatory therapies to prevent CVDs. However, at present, no canonical anti-inflammatory drug has a recommended indication for the treatment of T2D, despite the vast knowledge on the drivers and mechanisms of LGI in this setting.

Here, we build a framework to ideally depict a typical inflammatory trajectory of T2D, as compared with a healthy one. To this end, we summarize data reporting an alteration in the levels of circulating inflammatory markers at different stages of T2D, focusing on hs-CRP and IL-6, the most used markers to assess LGI. We encompass the whole trajectory of the disease by synthesizing data relative to the ability of inflammatory markers to predict incident T2D, the effect of glycemic control and other CVD risk factors on these same markers, the persistence of increased levels of LGI promoting CVD development despite risk factors control, and the ability of novel drugs to strongly curb the inflammatory trajectory of the disease, possibly by acting on the triggers of LGI. We finally anticipate the opportunity for a wider use of colchicine in people with T2D, once a reliable marker or a set of markers identifying subjects with a consistent LGI burden despite optimal polytherapy is established. In addition, for each stage of the hypothetical inflammatory trajectory, we also briefly describe the main molecular and cellular mechanisms held to underlie LGI in T2D.

## 2. Low-Grade Inflammation Precedes Diabetes Development

At the systemic level, IL-6 and CRP have been widely studied as markers of LGI in the context of diabetes because they are easily measurable inflammatory markers, widely correlated with the risk of developing the disease, and involved in the central biological processes of inflammation and insulin resistance [8]. From an analytical perspective, CRP has been very successful in providing a reliable, sensitive, and accessible measure of systemic inflammation. In particular, the development of high-sensitivity CRP can detect CRP levels as low as 0.1 mg/L and is essential in identifying chronic LGI and stratifying patients into different CV risk categories [9]. Many other molecules characterize LGI in T2D. For instance, the first evidence of inflammation in the adipose tissue of obese animals involved TNF-α [10], whose circulating levels are also associated with adiposity in humans [11]. However, we focused on hs-CRP and IL-6, since they are the most commonly used markers to assess LGI, and they both have a recognized diagnostic value for the detection of both T2D and CVDs [8,9,12,13].

The first strong evidence of LGI as a factor preceding the development of diabetes dates back to the early 2000s [14,15,16]. After that, several studies demonstrated the association of proinflammatory molecules with features of metabolic syndromes and clinically overt type 2 diabetes [17,18]. Wang and colleagues conducted a meta-analysis of prospective studies to determine whether elevated levels of inflammatory markers, specifically IL-6 and CRP, are associated with an increased risk of developing type 2 diabetes [12]. The study pooled data from 10 studies involving over 19,000 participants for IL-6 and 22 cohorts involving over 40,000 participants for CRP. The meta-analysis found a significant association between elevated IL-6 levels and an increased risk of type 2 diabetes, with a relative risk (RR) of 1.31. Similarly, elevated CRP levels were also associated with a higher risk, with an RR of 1.26. Noteworthily, the analysis highlighted a dose–response relationship, meaning that higher levels of these inflammatory markers corresponded with a progressively increased risk of developing type 2 diabetes. The results are consistent across various sensitivity analyses, which strengthened the evidence for these markers as predictors of diabetes.

The postulate that inflammation precedes the onset of diabetes is supported by findings indicating that individuals with pre-existing chronic inflammatory disorders are at an increased risk of developing T2D, and risk is associated with the severity of inflammation [19]. Indeed, one study used data from over 500,000 adults in the UK and found that both individuals with local organ-specific inflammatory disorders and with multisystemic chronic inflammation had a significantly higher risk of developing T2D, and higher CRP levels had a greater risk of multiple outcomes, i.e., T2D and CVDs [19].

Mechanistically, aging and obesity (or overnutrition) are the main triggers of LGI in T2D, being also the most relevant risk factors for the development of the disease. The term metaflammation has been coined to define the chronic LGI linked to metabolic diseases, often driven by unhealthy lifestyle factors like poor diet and physical inactivity [2]. Adipose tissue (AT) has so far been the focus of studies aimed at understanding metaflammation and the related molecular mechanisms. The onset of obesity, the major risk factor of T2D development, is determined by a prolonged energy imbalance, where caloric intake exceeds energy expenditure [20]. In this condition, the white adipose tissue (WAT) undergoes a series of structural and functional changes [21]. Adipocytes store excessive energy as triglycerides in one large lipid droplet, leading to a disproportionate increase in cell diameter (hypertrophy). Hypertrophic adipocytes secrete several factors, which modify the surrounding environment by promoting angiogenesis, remodeling of the extracellular matrix, and releasing proinflammatory molecules to promote WAT expansion [22]. This fosters hypoxia within the tissue, which further stimulates the release of inflammatory signals [23]. Among others, leptin, adiponectin, and IL-6 seem to play a key role in the initial response of AT to overnutrition [24,25,26,27]. The stress and inflammation in expanding adipose tissue attract monocytes from the peripheral blood and induce local proliferation of resident macrophages (adipose tissue macrophages, ATM) [28]. These macrophages polarize into a proinflammatory phenotype and may assume a typical crown-like structure that surrounds adipocytes when they are damaged or dead [29]. The presence of these proinflammatory macrophages in adipose tissue exacerbates local inflammation by releasing cytokines, which further perpetuate a cycle of inflammation and tissue stress. Cells of the acquired immunity are also recruited and stimulated [30,31], further contributing to this vicious cycle. The inflammatory cytokines released by various cell types of the WAT enter the bloodstream, leading to low-grade systemic inflammation [32]. As a result, TNF-α and IL-6 interfere with insulin receptor signaling by promoting the serine phosphorylation of insulin receptor substrate (IRS) proteins, which inhibits their activity [33]. As insulin signaling becomes less effective, glucose uptake by cells is reduced, and the liver’s glucose homeostasis becomes dysregulated [34].

Beyond the dysregulation of immune cells, the number of senescent cells within adipose tissue increases significantly in people with obesity and during aging [35]. Cellular senescence is a stereotyped response of multiple cell types to different stimuli, resulting in a stable cell cycle arrest and a range of phenotypic changes. Extensive replication, oncogenes, radiations, oxidative stress, and other insults are recognized triggers of senescence, which is characterized by a deep remodeling of the transcriptional and metabolic activity [36]. Senescent cells in adipose tissue actively contribute to a proinflammatory environment through the secretion of a variety of proinflammatory cytokines, chemokines, growth factors, and proteases, collectively known as the senescence-associated secretory phenotype (SASP). The SASP exacerbates local and systemic LGI [37]. WAT cells are highly prone to senescence due to both aging and obesity, regardless of chronological age [38]. The exact cell type(s) and the relative impact of each cell type (preadipocytes, macrophages, endothelial cells) contributing to WAT senescence are being explored. Senescent preadipocytes exhibit a well-defined phenotype characterized by a complex SASP, which contributes to local tissue inflammation and promotes macrophage infiltration, amplifying the immune response and perpetuating inflammation within the adipose tissue. Moreover, senescent preadipocytes show diminished capacity for both proliferation and differentiation, and their SASP further impairs the adipogenic potential of nearby progenitor cells, inducing senescence in them as well [39]. Macrophages with senescence features—beta-galactosidase positivity and p16 expression—release higher levels of proinflammatory cytokines and accumulate in the WAT of individuals with obesity. Interestingly, the distribution of these senescent cells varies between different fat depots (subcutaneous vs. visceral adipose tissue), with visceral fat showing a higher propensity for senescent cell accumulation and related inflammation [40]. In addition, even senescent endothelial cells have been found in AT from obese subjects [41].

The prominent role of senescence and SASP in AT dysfunction has been demonstrated by a study on mice. The clearance of p16-expressing cells, either genetically in mice or by treating senescent, apoptosis-resistant cells with senolytic drugs (drugs that selectively eliminate senescent cells) dasatinib (D) and quercetin (Q), enhanced the ability to generate new fat cells, reduced the enlargement of existing adipocytes, decreased macrophage infiltration, and significantly improved glucose regulation and insulin sensitivity [42]. Another study demonstrated that the intermittent clearance of cells that express high levels of p21 in adipose tissue of mice alleviates insulin resistance and that the selective inactivation of the NF-kB pathway within these cells is sufficient to obtain the same effect without killing the cells, supporting the role of SASP in this context. Additionally, the authors found that the senolytic drugs D and Q could reduce the presence of p21-high cells in human fat tissue and improve insulin sensitivity [43].

Beyond obesity, it is important to note that nonobese T2D patients also exhibit an elevated inflammatory score, which is determined by the average concentration of various cytokines in the bloodstream, including IL-6, IL-8, and TNFα [44]. Metaflammation shares many molecular features with ‘inflammaging’, i.e., the chronic, low-grade, systemic, inflammatory state associated with aging [45]. Levels of major circulating proinflammatory cytokines are increased in both conditions, thus prompting a combined causative effect of these two phenomena on diabetes. In general, excessive calorie intake and AT dysfunction should not be considered the only trigger and source of LGI in T2D [44,46].

In summary, overnutrition and aging promote a status of chronic LGI through multiple mechanisms involving AT, cells of innate and acquired immunity, senescent cells, and a large range of proinflammatory mediators. The resulting degree of LGI assessed by circulating biomarkers such as IL-6 and hs-CRP predicts the onset of T2D years before the development of overt hyperglycemia, sustaining its role in the pathogenesis of the disease.

## 3. Anti-Inflammatory Therapies Only Mildly Improve Glycemic Control

Given that LGI is a key driver of T2D, a number of trials have tested whether anti-inflammatory drugs could reduce the incidence of T2D in people with prediabetes or improve glycemic control in patients with frank disease. Most studies focused on drugs targeting the IL-1 pathway, considering its pivotal role in disease development [47].

A landmark study demonstrated the ability of the interleukin-1 antagonist anakinra to improve glycemic control. Indeed, after 13 weeks, HbA1c was 0.46 percentage points lower in the anakinra group compared with the placebo group, with a concomitant improvement in the levels of hs-CRP and IL-6 [48]. Of note, albeit modest, such a delta of HbA1c is not distant from that obtained with selected glucose-lowering drugs [49,50]. However, such an approach, i.e., targeting the IL-1 pathway, and in particular IL-1β, is not effective in reducing the progression of prediabetes to frank T2D. Indeed, a secondary analysis of the Cantos trial showed that despite consistent reductions in hs-CRP and IL-6, canakinumab did not reduce the incidence of new-onset diabetes in the subgroup of people with prediabetes [51]. Canakinumab lowered HbA1c during the first 6 to 9 months of treatment, but no long-term benefits on HbA1c or fasting plasma glucose were observed [51].

Similar data are available for salsalate, a prodrug of salicylate, an anti-inflammatory molecule that inhibits the cyclooxygenases and possibly the NF-κB pathway [52]. Several clinical trials consistently demonstrated an improvement in HbA1c or in other parameters of glycemic control with salsalate [53,54,55]. However, all these trials were of short duration, and no study specifically tested whether the use of this drug attenuates the progression of prediabetes to T2D, possibly also due to some side effects linked to the use of salsalate, an aspect that halted its further testing [3].

More recently, a secondary analysis of the LoDoCo 2 trial (see below) provided evidence for a numerically but not significantly lower incidence of T2D in patients with CVDs on daily therapy with low-dose colchicine [56], a drug that inhibits immune cells motility, mobilization, adhesion, and the secretion of proinflammatory factors [57]. More data are needed to evaluate the potential of such an approach.

Intuitively, canonical anti-inflammatory therapies attenuate LGI but do not remove or contrast the factors triggering or fueling inflammation. Thus, to observe a clinical benefit, continuous, long-term therapy is needed, which is often not possible with most anti-inflammatory drugs due to the intrinsically associated risks. Thus, when these drugs are discontinued and if the causes of LGI are not removed, the inflammatory trajectory of T2D progresses undisturbed [3].

## 4. Targeting Cardiovascular Risk Factors and Common Therapies Only Partially Attenuate Inflammation

Once established, T2D progresses unless the trigger(s) promoting its development is/are removed, e.g., a consistent weight loss after pharmacological treatment or bariatric surgery (see below). The canonical approach to treating T2D aims at targeting all the possible CVD risk factors, i.e., lifestyle, body weight, HbA1c, LDL cholesterol, blood pressure, smoking, and renal function [58].

Glycemic control, measured as HbA1c, is associated with the degree of activation of immune cells of both the innate [59,60] and the acquired [61,62] immunity, as well as the circulating levels of hs-CRP and IL-6 [63,64,65,66]. For instance, patients with good glycemic control, i.e., HbA1c < 7%, have lower circulating levels of IL-6 [66] but also a less impaired immune response after vaccination [62]. Coherently, most glucose-lowering drugs are accompanied by some degree of an anti-inflammatory effect [3]. Indeed, even “older” drugs such as metformin, acarbose, and the PPARℽ activators glitazones are accompanied by an amelioration of selected markers of LGI at the systemic level [67,68,69]. While most of the anti-inflammatory effect might be ascribed to an improvement in glycemic control, metformin and glitazones are held to possess intrinsic anti-inflammatory properties [70,71,72]. Such data are not available or less consistent for sulfonylureas [73], a finding in line with the postulate that improving glycemic control by increasing insulin levels is not associated with an anti-inflammatory effect [74,75]. Of note, sulfonylureas, which are not accompanied by a clear CVD benefit, are progressively becoming the last option for the treatment of T2D according to common algorithms [58,76].

Despite the widespread knowledge relative to the causal role of LDL cholesterol in promoting inflammatory responses within the vascular wall and the subsequent development of atherosclerosis [77], the relationship between LDL cholesterol and systemic LGI is not linear. Indeed, even though the circulating levels of oxidized LDL are directly correlated with the levels of hs-CRP [78], the same does not apply to the relationship between LDL cholesterol and hs-CRP [79], which appears to be J-shaped when testing the general population [80]. In this respect, historical studies in the field suggest that hs-CRP and LDL-cholesterol are both associated with CVD development, but there is a weak correlation between them [79,81]. Of note, this is of clinical relevance since people with low LDL-cholesterol levels can still have a consistent CVD risk driven by high levels of hs-CRP, a phenomenon referred to as residual inflammatory risk [82], which is detailed below. Despite the lack of a clear linear relationship between LDL cholesterol and hs-CRP (or even IL-6), a minimum threshold of LDL cholesterol lowering to obtain a significant reduction in hs-CRP levels has been suggested [83]. Almost all the existing lipid-lowering drugs have some degree of anti-inflammatory effects, with statins being the most studied but possibly also the most potent [84]. Indeed, statins, ezetimibe, and bempedoic acid all have data supporting their ability to lower circulating levels of hs-CRP but also IL-6 [84,85]. Of note, the anti-inflammatory effect is not proportional to the levels of LDL-cholesterol achieved [79,84], suggesting that other mechanisms beyond LDL cholesterol lowering concurs with the anti-inflammatory effect of these drugs. In addition, the use of potent LDL-lowering drugs such as PCSK9 inhibitors is not associated with hs-CRP reduction [84], while these same drugs have been suggested to present LDL cholesterol-independent, anti-inflammatory activity within the vascular wall [86,87]. However, notwithstanding all these therapeutic options, recent real-life data suggest that the proportion of patients with an increased burden of LGI despite optimal lipid-lowering therapy, i.e., with LDL cholesterol < 70 mg/dL, is consistent [82].

A number of studies demonstrated that high levels of hs-CRP and IL-6 are associated with high blood pressure, independently of diabetes [88,89]. Most commonly used antihypertensive therapies are associated with a reduction in the circulating levels of hs-CRP and IL-6 [90,91]. Similarly to what has been reported with lipid-lowering therapies, the degree of hs-CRP reduction with antihypertensive therapies does not appear to be proportional to the achieved levels of blood pressure [92]. One of the hypotheses is that an effective anti-inflammatory treatment is obtained only with angiotensin-converting enzyme inhibitors or angiotensin-receptor blockers, since angiotensin II is a recognized proinflammatory peptide [90,93]. However, even with these drugs, the effect on these markers is modest [92,94]; thus, patients on optimal antihypertensive therapy still have a consistent LGI burden.

In summary, targeting multiple CVD risk factors with commonly used drugs in T2D is associated with a partial improvement in LGI. Such amelioration is proportional to the levels achieved for HbA1c [95] but not for LDL cholesterol [84] and blood pressure [92]. More importantly, none of these therapies completely suppress LGI, possibly because none of them actually impact the roots of inflammation, i.e., the pathways instigated by obesity (or overnutrition) and aging, nor the long-lasting mechanisms instigated by periods of exposure to uncontrolled risk factors (see below), with the possible exception of metformin [71,96]. Thus, targeting CVD risk factors partially improves but does not curb the inflammatory trajectory of T2D, which progresses despite optimal polytherapy in most people with this condition.

## 5. Residual Inflammatory Risk Predicts Cardiovascular Diseases

The concept of residual inflammatory risk emerged from research into cardiovascular disease, highlighting that even when traditional risk factors like LDL cholesterol are controlled, patients may still face significant risk due to underlying inflammation. This idea gained attention after seminal studies like the one by Ridker et al. in 1997 [97], who demonstrated that elevated levels of C-reactive protein (CRP) were associated with an increased risk of future cardiovascular events, independent of lipid levels. Interestingly, it soon appeared evident that the prognostic value of CRP was particularly evident in patients with lower levels of other conventional determinants of risk [98]. Moreover, despite effective lifestyle and pharmacological interventions targeting LDL-C, the incidence of adverse cardiovascular events is growing, also among subjects with well-controlled LDL-C [99]. These observations suggested that inflammation plays a critical role in cardiovascular disease, leading to a deeper investigation into anti-inflammatory strategies as potential therapeutic interventions. This paradigm shift underscored the importance of addressing inflammation as a distinct target for reducing cardiovascular risk beyond cholesterol management alone [100]. However, targeting inflammation in patients with atherosclerotic CVDs has proven difficult due to the high biological variability in systemic inflammatory responses, which are driven by numerous pathways, each influencing the inflammatory response at different times and with varying intensity across different patients. Although a multitude of inflammatory mediators have been assessed, hs-CRP, due to its wide availability and analytical standardization, has emerged as the preferred biomarker of residual inflammatory risk [101], despite the knowledge relative to the inability of this marker to intercept all the existing inflammatory phenomena [102]. Interestingly, hs-CRP has proved valuable in both primary [103] and secondary [104] prevention, helping to identify the role of pre-existing inflammation in the development of atherosclerotic CVD and the extent of inflammatory responses following MACE. Despite its inclusion in the American Heart Association’s guidelines for primary cardiovascular prevention [105], the use of hs-CRP remains limited in European countries, primarily due to concerns about the lack of specific treatment options for patients with elevated hs-CRP levels [106]. Nevertheless, the JUPITER trial provided convincing evidence that the use of statins confers benefits in terms of CV risk that are not fully explained by LDL-C reduction, such as those observed in patients with residual inflammatory risk (hs-CRP > 2 mg/L) but on-target LDL-C levels [107]. These results led to the conceptualization of a treatment strategy that relies on lifestyle interventions and statin therapy for all patients and on a more personalized approach aimed at reducing either residual cholesterol or inflammatory risk with specific treatments, such as PCSK9 and IL-1b inhibitors, respectively [106].

The success of the CANTOS [4], COLCOT [5], LoDoCo [6], and LoDoCo 2 [7] trials has finally demonstrated that targeting inflammation is a suitable strategy to reduce CVD risk. Of note, while the first two trials adopted a patient enrichment strategy, the LoDoCo trials simply enrolled patients with chronic coronary disease. The CANTOS trial only included patients with hs-CRP levels above 2 mg/L, while the COLCOT trial focused on patients with systemic inflammatory activation shortly after (<30 days) atherosclerotic CVD events. These strategies were key in demonstrating the effectiveness of the IL-1β inhibitor canakinumab and colchicine, respectively, in preventing the recurrence of cardiovascular events. On the contrary, the LoDoCo trials provided evidence of CVD benefit for low-dose colchicine, even in patients with chronic CVDs and without any screening for hs-CRP. Among the patients enrolled in all these trials, a consistent number of subjects had T2D or prediabetes, which already represents a condition associated with increased LGI [51].

Given the key role of LGI in T2D, people with T2D are obvious candidates to harbor residual inflammatory risk. We previously reported that a consistent proportion of subjects with T2D have residual inflammatory risk despite optimal LDL-C control [108]. Interestingly, levels of hs-CRP were related not only to blood lipids but also to variables describing central obesity, insulin resistance, and poor glycemic control, especially in patients with on-target LDL-C, suggesting a contribution of multiple risk factors beyond dyslipidemia to the prevalence of residual inflammatory risk. Notably, the independent prognostic value of hs-CRP for cardiovascular and all-cause mortality was also confirmed in T2D [109,110], highlighting that systemic inflammation should be targeted and monitored with the same importance as blood glucose and lipid levels.

Understanding the factors contributing to residual inflammatory risk in type 2 diabetes (T2D) remains incomplete. A key observation is that the level of systemic inflammation following a major adverse cardiovascular event (MACE), such as a myocardial infarction (MI), is only partially linked to the extent of myocardial damage [111]. This implies that other risk factors, including prior exposures and genetic or epigenetic predispositions, may play a role. Randomized and observational studies have identified associations between residual inflammatory risk and several risk factors, including sex [112], abdominal obesity [113], blood glucose levels [108], lifestyle [114], the use of T2D medications [115,116], and presence of comorbidities [20]. These factors appear to converge on selected shared pathways, ultimately leading to the development of chronic LGI [117].

Beyond the contribution provided by undruggable or often untargeted risk factors/conditions, e.g., sex, lifestyle, obesity, and nephropathy, periods of exposure to uncontrolled risk factors might also contribute to residual inflammatory risk in T2D [118,119]. Indeed, hyperglycemia (and possibly dyslipidemia) triggers a large range of enduring changes and the long-term activation of pathways and mechanisms sustaining LGI chronically, e.g., long-lasting oxidative stress, mitochondrial dysfunction, permanent epigenetic alterations, changes in the circulating levels of extracellular vesicles and shuttled molecules, senescent cells accumulation, mobilization and stimulation of immune cells, and trained immunity [118,120,121,122,123,124,125,126,127,128,129,130,131,132,133,134,135]. For instance, hyperglycemia triggers a range of epigenetic modifications, e.g., specific DNA methylations and histone modifications, mostly underlying a chronic expression of proinflammatory genes in immune cells and possibly providing the mechanistic rationale explaining the phenomenon of metabolic memory, i.e., the persisting risk of complications provided by periods of exposure to hyperglycemia despite subsequent glucose normalization [118,119,128]. More recently, the accrual of senescent cells has been recognized as a risk factor for the development of both T2D and CVDs [136]. Multiple reports evidenced the presence of senescent cells in virtually all diabetes-relevant organs, i.e., adipose tissue, muscles, liver, and the pancreas [137,138,139,140]. Of note, the selective removal of senescent cells with specific drugs promoting their clearance, termed senolytics, is associated with a range of metabolic and other benefits in animal models of diabetes [42,141]. Furthermore, not only the presence of senescent cells within the atherosclerotic plaque has been associated with T2D-related CVDs [142] but a faster rate of cellular senescence within other tissues [143,144]—measurable directly or through proxy indicators like leukocyte telomere length—has also been related to T2D clinical characteristics and outcomes. This suggests that the senescence-associated secretory phenotype (SASP), which includes inflammatory cytokines and other nonprotein mediators like nucleic acids, is a major contributor to residual inflammatory risk. Importantly, several lifestyle factors, including sleep patterns [145], physical inactivity [146], and overfeeding [147], can accelerate senescence either directly or indirectly through the modulation of gut microbiota and the epigenetic makeup [71,148]. Parallel to this, exposure to external stressors may trigger maladaptive mechanisms, which include the so-called trained immunity, i.e., a reprogramming of innate immune cells that allows for stronger and prompter responses towards pathogens but results in accelerated atherosclerosis progression when triggered by harmful stimuli like atherogenic lipoproteins and oxidative stress [149].

Beyond the inflammatory pathways instigated by hyperglycemia and other risk factors, one of the theories explaining an increased burden of LGI in T2D is the faster pace of aging observed in this population, which could be either intrinsic or driven by the disease itself [44,46,118,137,150,151,152]. In a recent study, we observed significant differences in the genome-wide methylation of peripheral blood mononuclear cells (PBMCs) between patients with T2D who survived and those who died over a follow-up period of more than 15 years, even when matched for major confounders [153]. Additionally, we found that epigenetic age, as measured by methylation clocks, can independently predict all-cause mortality in these patients. Notably, deceased patients had higher methylation-predicted levels of C-reactive protein (CRP) and exhausted CD8+ T cells, indicating an epigenetic trigger for residual inflammatory risk.

A number of novel approaches are trying to target the mechanisms governing senescent cell accumulation, epigenetic changes, trained immunity, and other pathways sustaining LGI in different diseases and conditions [154]. Among the most notable are phase 1 and 2 studies on the senolytic drugs dasatinib and quercetin, which have shown effectiveness in clearing senescent cells in humans with diabetic kidney disease, an effect associated with an attenuated burden of LGI [155]. However, none of these approaches has yet reached the clinical stage. Thus, the pathways fueling LGI remain largely untargeted in patients with T2D.

## 6. Disease-Modifying Drugs Could Curb the Inflammatory Trajectory of Diabetes

The landscape of diabetes treatment has changed in the last fifteen years. Since the introduction of cardiovascular outcome trials, along with other trials tailored to assess the effect of novel drugs on heart failure and renal outcomes, it is increasingly evident that sodium–glucose co-transporter 2 (SGLT-2) inhibitors (i) and glucagon-like peptide-1 receptor agonists (GLP-1RA) dramatically improve patients’ cardiovascular and renal outcomes. In particular, selected GLP-1RA reduces the incidence of atherosclerotic endpoints in patients with T2D, halts the progression of heart failure with preserved ejection fraction in obese patients with or without T2D, and also demonstrates favorable renal outcomes in patients with T2D [156,157,158,159]. Similarly, SGLT-2i has a proven benefit on major adverse cardiovascular events in T2D, ameliorating the progression of nephropathy in a range of patients, irrespective of diabetes status, renal impairment, and the presence/absence of heart failure, and reducing heart failure events and cardiovascular death in patients with heart failure, T2D, chronic kidney disease, and atherosclerotic CVDs [157,160,161]. Of note, preliminary observational studies suggest that the benefit of these drugs might be additive or even synergic when used in combination [162,163]. Such an ability to prevent the development of multiple complications, coupled with the observation that such benefit seems independent from the effect of these drugs on canonical risk factors [164], has identified these medications as disease-modifying drugs [165].

Both GLP-1RA and SGLT-2i have a powerful effect against LGI. Indeed, recent trials testing the effect of GLP-1RA in obese people with heart failure with preserved ejection fraction, with or without T2D, showed that these drugs are accompanied by a dramatic reduction in the levels of hs-CRP [158,159]. Powerful effects are also observed on other inflammatory mediators, including IL-6 [166]. Similar data are available for SGLT-2i, which ameliorates the circulating levels of multiple inflammatory markers in diverse settings [167,168,169,170].

Mechanistically, even though selected reports suggest a direct anti-inflammatory effect of these drugs on immune or other proinflammatory cells [167,171], it is increasingly evident that GLP-1RA and SGLT-2i act on the pathogenesis of cardiometabolic diseases, or at least on selected features promoting T2D and LGI. Indeed, GLP-1RA suppresses appetite, thus counteracting overnutrition and leading to impressive weight loss in people with obesity [172,173]. The removal of overnutrition and obesity obviously translates into a powerful curbing of the inflammatory trajectory of T2D, possibly halting its development. Indeed, even though the definition of diabetes remission is an ongoing debate [174], available evidence suggests that GLP-1RA, but especially tirzepatide, a dual GLP-1/GIP agonist with potent weight-reducing properties, is able to induce glycemic control compatible with that of people with normoglycemia in a large proportion of patients with T2D [175,176,177]. In the multiple SURPASS trials, 43 to 62% of participants achieved HbA1c < 5.7%, despite such a glycemic target not being a prespecified goal [178]. Of note, people achieving normoglycemia were younger and had a shorter duration of diabetes [178], an observation compatible with the postulate that early intervention on the inflammatory trajectory of T2D might promote its curbing to healthy levels, preventing the development of the disease.

Beyond suppressing appetite, another approach to promote caloric restriction, an intervention with an established broad benefit [179,180], is to promote the elimination of calories. SGLT-2i is a class of drugs that reduce glucose levels by enhancing its excretion through urine, leading to a net loss of calories. This process triggers a systemic metabolic shift that encourages the use of ketones and fatty acids as alternative energy sources to glucose [181,182]. As a result, these drugs modulate key nutrient-sensing pathways, such as mTOR and the inflammasome, which are held to drive T2D development and, more broadly, aging [183,184,185]. Thus, early use of SGLT-2i is expected to be accompanied by a broad and long-lasting benefit on LGI, given the ability of these drugs to counteract key drivers of inflammation and T2D. Even though there is no evidence that SGLT-2i promotes diabetes remission at present, SGLT-2i is demonstrated to reduce the incidence of T2D [186] and to attenuate the deleterious effects of poor glycemic control on CVDs in patients with a recent T2D diagnosis, an effect observed only when the drug is introduced early [187].

Overall, these data clearly evidence the ability of GLP-1RA and SGLT-2i to curb the inflammatory trajectory of T2D by acting on the factors and/or pathways triggering or sustaining LGI. Their stunning benefit on a large range of outcomes, which possibly includes diabetes remission or prevention, suggests that an early introduction of these drugs during the course of T2D could maximize their benefit and change the trajectory of the disease [165,188,189].

## 7. Targeting Low-Grade Inflammation with Colchicine for CVD Prevention in Diabetes

As mentioned above, the CANTOS [4], COLCOT [5], LoDoCo [6], and LoDoCo 2 [7] trials all enrolled a consistent number of subjects with prediabetes or T2D. A subgroup analysis comparing the incidence of cardiovascular outcomes in patients with vs. without T2D in the Cantos trial revealed that the effect of canakinumab was not different in these two groups, being equally protective against MACE [51]. However, almost all (i.e., ~83%) of the participants without T2D had prediabetes, which, as explained above, is already a condition with an increased LGI. Thus, it is reasonable that the benefit of the anti-inflammatory intervention is comparable in the two groups. In the COLCOT trial, which enrolled patients with a recent MI, the prevalence of T2D was lower, i.e., ~20%, but the presence of prediabetes was not explored [5]. A recent secondary analysis in the subgroup of patients with T2D revealed that colchicine, at 0.5 mg daily, leads to a large reduction in cardiovascular events in this population [190]. Similar data have been provided by the LoDoCo 2 trial. Even in this trial, the prevalence of T2D was roughly 20%, and prediabetes was not considered [7]. A subgroup analysis demonstrated that the effect of colchicine on the incidence of cardiovascular outcomes was not different in patients with vs. without T2D, with the drug being equally effective in preventing MACE independently of diabetes [56]. If the groups of subjects without T2D included people with prediabetes, it is reasonable to expect a comparable degree of cardiovascular benefit provided by colchicine.

Another anti-inflammatory drug that is being tested for its potential to decrease the incidence of CVD is the anti-IL-6 antagonist ziltivekimab. Indeed, a recent phase 2 trial demonstrated that this drug markedly reduced markers of LGI and thrombosis in people with chronic kidney disease and hs-CRP > 2 mg/L [191]. Of note, in this preliminary trial, ~70% of enrolled individuals had T2D. Thus, the planned large-scale CVOT of ziltivekimab conducted among patients with chronic kidney disease, increased CRP, and established CVDs will likely include a large proportion of people with T2D.

Beyond the scientific interest in determining whether colchicine or other anti-inflammatory drugs are more effective in preventing CVD in the T2D population compared with people without metabolic diseases, it is clear that individuals with prediabetes and T2D are the ideal candidates for such treatments. As explained above, the inflammatory trajectory of T2D deviates early from the ideal one, is driven by a complex series of mechanisms and pathways, is progressive, and remains largely untargeted with canonical therapies, with the possible exception of early intervention with disease-modifying drugs. Indeed, in the real world, a large proportion of patients with T2D present with evidence of residual inflammatory risk. In addition, hs-CRP misses some components of LGI, an aspect that might hide some patients potentially benefitting from anti-inflammatory therapies. While canakinumab did not receive approval for the prevention of CVD due to the potential risk of fatal infection [192], the FDA approved the use of low-dose colchicine to reduce the risk of myocardial infarction, stroke, coronary revascularization, and cardiovascular death in people with established atherosclerotic disease or with multiple risk factors for CVDs [193]. While T2D is commonly considered just one of such risk factors, knowledge derived from clinical and basic research suggests that almost all subjects with T2D have an increased degree of LGI and thus will likely benefit from the use of an anti-inflammatory drug in primary prevention as well, and not only after a diagnosis of atherosclerotic CVD or after an acute MI. Overweight, unhealthy lifestyle, smoking, diabetes duration, diabetes complications, poor glycemic control, and other factors are all associated with a consistent inflammatory burden. Ideally, a reliable marker or a set of markers of LGI (including or not hs-CRP) should be available to guide treatment decisions and evaluate the opportunity of treating individuals at high CVD risk with colchicine. Until such marker(s) are available and their role for treatment selection is established, we propose that colchicine should be used in a large range of individuals with T2D, i.e., all except those without all the characteristics listed above and thus young, lean, nonsmokers, with short disease duration, good glycemic control, and no complications.

**Figure 1:**
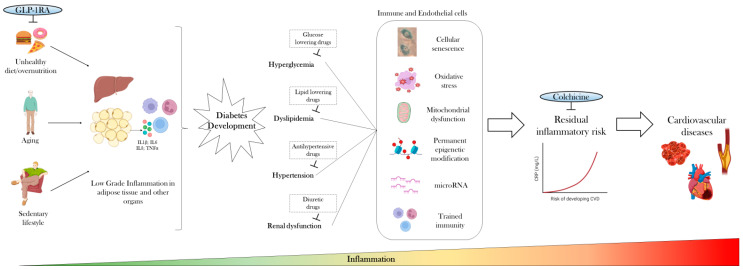
**Trigger mechanisms sustaining inflammation in T2D.** Aging, overnutrition, and a sedentary lifestyle promote early inflammation through multiple mechanisms involving the adipose tissue and other diabetes-relevant organs. After T2D development, the canonical risk factors for CVD typical of T2D, e.g., hyperglycemia, promote the activation of a range of long-lasting mechanisms and pathways chronically fueling LGI even after normalization of such risk factors. The resulting proinflammatory program might underly the so-called residual inflammatory risk, the persistent elevation of hs-CRP despite LDL cholesterol control, which represents a risk factor for CVD development. This deleterious progression of inflammation might be targeted both early by introducing disease-modifying drugs such as GLP-1RA and late by treating residual inflammatory risk with colchicine.

The mechanisms underlying LGI during the whole trajectory of T2D are briefly summarized in Figure 1, which emphasizes the novel opportunities provided by disease-modifying drugs such as GLP-1RA to intercept early inflammation and by colchicine to treat LGI late during the course of T2D.

## 8. Conclusions and Future Directions

**Figure 2:**
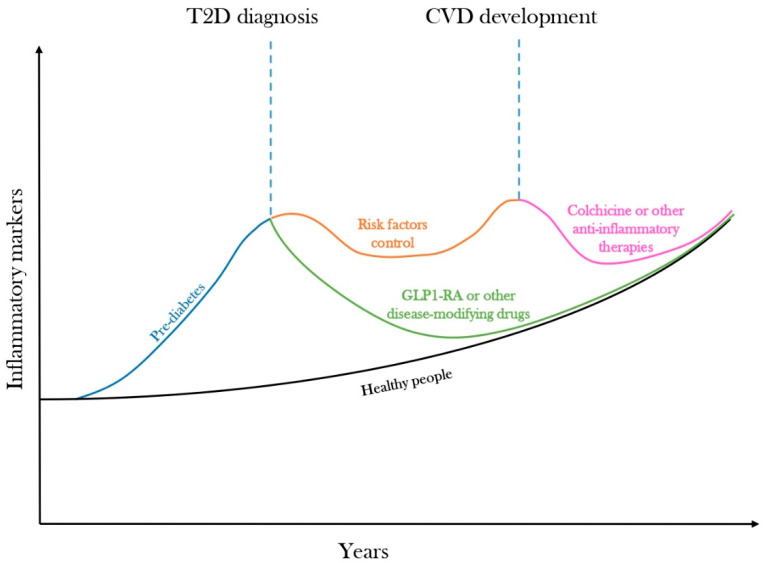
**The inflammatory trajectory of type 2 diabetes.** A postulated hypothetical trajectory of low-grade inflammation (LGI) in type 2 diabetes (T2D), which deviates early from a healthy status (black line) years before T2D development, when people are often characterized by prediabetes or other metabolic abnormalities (blue line). When T2D is diagnosed, individuals are usually treated to reach specific goals for multiple CVD risk factors, but this approach only partially attenuates LGI (orange line) since the triggers of LGI are not removed, and many long-lasting inflammatory mechanisms are unaffected by conventional therapies. If left undisturbed, LGI will promote cardiovascular disease (CVD) development, but this could still be attenuated by the use of colchicine or other canonical anti-inflammatory therapies with proven benefits for CVDs (pink line). Alternately, an early interception of T2D with a prompt introduction of disease-modifying drugs, such as GLP-1RA, which remove overnutrition and obesity and thus key triggers of LGI, might allow an efficient curbing of the inflammatory trajectory (green line), possibly folding it to a healthy status and avoiding the noxious long-term consequences of the disease. Further studies are needed to clarify this latter aspect.

Here, we depicted a hypothetical inflammatory trajectory for T2D (graphical summary postulated in Figure 2). LGI is a critical contributor to the onset and progression of T2D and associated CVD. The inflammatory trajectory in T2D diverges early from a healthy state and remains elevated even after diagnosis and treatment. Factors such as excessive caloric intake, poor dietary habits, physical inactivity, obesity, and aging are well-established triggers of LGI. Even before the full clinical manifestation of the disease, key CVD risk factors activate various inflammatory pathways and mechanisms, including immune cell activation, the accumulation of senescent cells, persistent epigenetic modifications, and trained immunity, all of which contribute to the chronic maintenance of LGI. While targeting these risk factors can partially alleviate LGI, certain long-lasting inflammatory pathways remain unaffected by conventional therapies, leading to a continued increased burden of LGI in many T2D patients, which, as a result, often have evidence of residual inflammatory risk.

At present, there might be two possible options to target LGI in T2D. GLP-1RA and SGLT-2i appear to influence the underlying pathogenesis of inflammation and T2D, potentially eliminating LGI triggers and preventing the disease. The early use of these drugs or powerful lifestyle interventions might allow an early curbing of the pathological inflammatory trajectory before the onset of T2D and should be the preferred strategy. Alternatively, in the case of patients with established disease and an increased LGI despite the proper targeting of multiple CVD risk factors, the only option is to cope with inflammation, trying to attenuate it or limiting its deleterious consequences on the vasculature using colchicine or eventual, future anti-inflammatory drugs with proven clinical benefit. Additional studies are needed to establish whether the early use of GLP-1RA or other drugs can reverse T2D and whether anti-inflammatory therapies should be broadly used in individuals with this condition.

## Data Availability

No data were generated or analyzed for or in support of this paper.
